# A Systematic Review and Meta-Analysis on the Use of Rivaroxaban as Thromboprophylaxis Following Bariatric Surgery

**DOI:** 10.1007/s11695-026-08774-3

**Published:** 2026-07-01

**Authors:** Sarah Catania, Clifford Caruana

**Affiliations:** 1 Edinburgh Surgery Online programme ChM (Masters in Surgery) in General Surgery offered jointly by the Royal College of Surgeons of Edinburgh and the University of Edinburgh, University of Edinburgh, Edinburgh, United Kingdom; 2 Department of General Surgery, Mater Dei Hospital, Imsida, Malta; 3 Department of General Surgery, Mater Dei Hospital, Imsida, Malta

**Keywords:** Rivaroxaban, Thromboprophylaxis, Bariatric surgery, Venous thromboembolism, Bleeding

## Abstract

Bariatric surgery and obesity increase VTE risk. While VTE incidence after bariatric surgery is low, it impacts morbidity and 30-day mortality. The lack of consensus on the optimal thromboprophylaxis has driven research into alternatives like DOACs. The objective of this study is to determine the efficacy and safety of rivaroxaban as thromboprophylaxis after bariatric surgery and the most effective thromboprophylaxis strategy. Findings from the nine included studies show that rivaroxaban is as effective as controls for DVT/PE prevention, with no PMVT cases. The incidence of minor and major bleeding requiring transfusion are more common with rivaroxaban however the meta-analysis does not consider this as significant evidence against its use. In the subgroup analysis, no significant differences are observed between long- and short-course rivaroxaban regimens across all primary outcomes, though shorter courses showed fewer bleeding complications.

## Key points

- Rivaroxaban appears to be a safe and effective thromboprophylactic option afterbariatric surgery.
- Short courses are suitable for most patients and extended use should be reserved for high-risk cases following anindividualised risk assessment.
- Determining the optimal thromboprophylaxis regimen remains a significant challenge.

## Introduction

The global rise in severe obesity has led to an increase in the number of patients referred for bariatric surgery. Both severe obesity and bariatric surgery are recognised risk factors for developing venous thromboembolism (VTE). Although the incidence of VTE namely deep vein thrombosis (DVT) and pulmonary embolism (PE), is relatively low after bariatric surgery, ranging from 0.2% to 5%, it is associated with significant morbidity and contributes to 30-day mortality, accounting for one in five deaths. Overall, VTE rates are comparable between sleeve gastrectomy (SG) and Roux-en-Y gastric bypass (RYGB) however, portomesenteric vein thrombosis (PMVT), which occurs in 0.3–1.8% of cases, is more commonly associated with sleeve gastrectomies than other bariatric procedures. PMVT tends to present late, with a median symptom onset of 14 days. Post-surgical bleeding following bariatric surgery occurs at a rate of 0% to 6%. VTE following bariatric surgery has been associated with several risk factors, including age over 50 years, a history of smoking, prior VTE events, and postoperative complications such as anastomotic leaks [18, 19]. According to the American Society for Metabolic and Bariatric Surgery (ASMBS) position statement on perioperative venous thromboembolism prophylaxis in bariatric surgery, most bariatric surgery patients are at least at moderate VTE risk due to obesity, laparoscopic procedures, and perioperative immobility. Some may be at high or very high risk, depending on individual and surgical factors [1].

According to clinical guidelines, pharmacological thromboprophylaxis with low molecular weight heparin (LMWH), combined with early mobilisation and the use of intermittent pneumatic compression stockings, are sufficient measures to prevent VTE. However, these practices are not uniformly applied worldwide, and there is no consensus on the most effective thromboprophylaxis strategy. LMWH is currently the most widely adopted agent for extended prophylaxis due to its efficacy, although its cost and requirement for subcutaneous administration may limit its practicality, especially in resource-constrained settings [19].

The most recent European guidelines published in 2024, suggest that in patients at high risk of VTE with low bleeding risk, pharmacological prophylaxis with LMWH, UFH, or fondaparinux is strongly recommended over no prophylaxis. DOACs are not currently recommended in the European guidelines primarily due to the limited clinical evidence supporting their use in this context, as well as concerns regarding potential malabsorption, particularly following procedures such as RYGB, which may significantly alter drug absorption and efficacy. For individuals with a body mass index (BMI) > 40 kg/m^2^ or weight > 150 kg, higher doses of these agents are suggested over standard doses. Routine monitoring of anti-Xa levels is not advised. Combined pharmacological and mechanical prophylaxis is recommended, along with extended prophylaxis for at least 10 days post-operatively, as up to 70 to 80% of VTE events occur after hospital discharge [2].

In line with this, in the International Society on Thrombosis and Haemostasis (ISTH) guidelines, DOACs are reported as not being favoured in the acute setting post-bariatric surgery due to malabsorption risks, and are not recommended in patients with a BMI > 40 kg/m^2^ or a weight > 120 kg because of the lack of available clinical data, and if to be used, drug specific peak and trough levels need to be monitored.

The use of DOACs, including rivaroxaban has increased in recent years, primarily due to their favourable safety and efficacy profiles, oral administration, fixed dosing regimen, low potential for drug interactions, and lack of food-related interactions, all of which enhance patient compliance. It has a rapid onset of action, is a long-acting anticoagulant with an oral bioavailability of 80–100% with a 10 mg dose over 24 h and reaches peak plasma levels within two to four hours. Absorbed mainly in the stomach, clinical trials suggest that the anatomical and absorption changes brought about by bariatric surgery and weight loss do not impact the pharmacokinetics and pharmacodynamics of the drug [5], [6], [11].

Rivaroxaban is approved for primary VTE prevention, and considered appropriate for use regardless of BMI or body weight in patients undergoing elective hip or knee arthroplasty [13]. Studies began to explore the use of rivaroxaban following bariatric surgery, especially since a high BMI in orthopaedic patients did not impact the drug’s effectiveness. A meta-analysis of 5,101 patients found no significant difference in VTE or bleeding risk between rivaroxaban and LMWH after non-major orthopaedic surgery [21]. Similarly, a post hoc RECORD analysis, found no outcome differences between patients with BMI ≥ 40 kg/m2 and those with BMI < 40 kg/m2 receiving rivaroxaban or enoxaparin after elective THA or TKA [9].

Rivaroxaban shows consistent pharmacokinetics and pharmacodynamics in obese patients. The RECORD trials demonstrated superior VTE prevention vs enoxaparin, with similar early bleeding rates, though bleeding was higher with rivaroxaban over time in those > 90 kg. Routine monitoring is not needed, as per Moore and Kröll [15], despite 2016 ISTH guidance, and obesity appears to have minimal impact on its efficacy or safety. This aligns with findings from another review indicating that obesity does not significantly affect rivaroxaban’s pharmacology, efficacy, or safety profile [3].

Bariatric surgery can alter the pharmacokinetics of orally administered drugs due to the postoperative anatomical and physiological changes. These include accelerated gastric emptying, reduced transit time through the small intestine, decreased absorptive surface area, and alterations in gastric pH. As a result, malabsorption is a well recognised postoperative concern, with deficiencies in fat-soluble vitamins such as vitamin K frequently reported following procedures like RYGB. Rivaroxaban was the first DOAC to have its pharmacokinetic profile specifically evaluated in the context of bariatric surgery. Current evidence suggests that a 10 mg dose of rivaroxaban maintains stable pharmacokinetics and pharmacodynamics postoperatively, with no clinically significant impact necessitating routine dose adjustment. These findings were supported by a follow-up extension study conducted six to eight months after surgery, reinforcing the drug’s stability in this patient population. Nonetheless, evidence remains limited, and further clinical research is required to establish the safety and efficacy of rivaroxaban in individuals undergoing bariatric procedures [3], [15].

Questions remain regarding whether dosing should be adjusted based on the patient’s weight and whether a short (up to 14 days) or long course (up to 30 days) of treatment should be administered, particularly given that over 70% of VTE cases occur after discharge, within the first 30 days post-surgery. Given that the majority of VTE incidents occur after discharge, extended-duration thromboprophylaxis is particularly important in patients at elevated risk [5], [11], [19]. The aim of this systematic review and meta-analysis is to determine the efficacy and safety of rivaroxaban as thromboprophylaxis after bariatric surgery, and to determine the most effective thromboprophylaxis strategy.

## Methods

The protocol was registered on the PROSPERO database. The systematic review was performed according to the PRISMA (Preferred Reporting Items for Systematic reviews and Meta-Analyses) guidelines.

The eligibility criteria for this review were established using the PICO framework. The population includes patients undergoing bariatric surgery, namely SG, RYGB, One Anastomosis Gastric Bypass (OAGB), revisional procedures, biliopancreatic diversion, via open, laparoscopic or robotic approaches. The intervention involves administration of a daily dose of rivaroxaban (10 mg in the majority of patients) for a duration of up to 30 days postoperatively. Comparison is made with a subgroup of patients, in which the control arm received LMWH or UFH, while the remaining patients do not have a comparison control arm. The primary outcomes include assessing the incidence of symptomatic or asymptomatic DVT, PE and PMVT. Secondary outcomes include evaluating the occurrence of postoperative bleeding complications, classified as minor, moderate, or major requiring transfusion or surgical intervention.

The inclusion criteria include RCTs, prospective and retrospective cohort studies, available in open access and published in the English language with available abstracts. Studies to be included report on the incidence of DVT, PE, and/ or PMVT and potentially the bleeding risk of individuals undergoing any form of bariatric surgery with rivaroxaban specified as the anticoagulant of choice, dosages, initiation timing, and duration of therapy. The exclusion criteria include literature reviews, case studies, expert opinions. Clinical trials which are still in progress and incomplete are also excluded.

A comprehensive literature search was conducted using Google Scholar, PubMed, and the Cochrane Library to identify relevant studies published between 2019 and 2024. The date range was chosen to capture the most recent evidence on the use of DOACs, namely rivaroxaban, in the context of bariatric surgery. A combination of keywords was used in the search strategy, including: “rivaroxaban,” “thromboprophylaxis,” “thromboemboli$,” “thrombosis,” “prophylaxis,” and “bariatric surgery.” The search was restricted to studies published in English and no other filters were applied. All retrieved references were managed using Zotero (version 7.0.15). Data was extracted independently by one reviewer and independently verified by another reviewer using a pre-formatted Microsoft Excel spreadsheet.

**Fig. 1:**
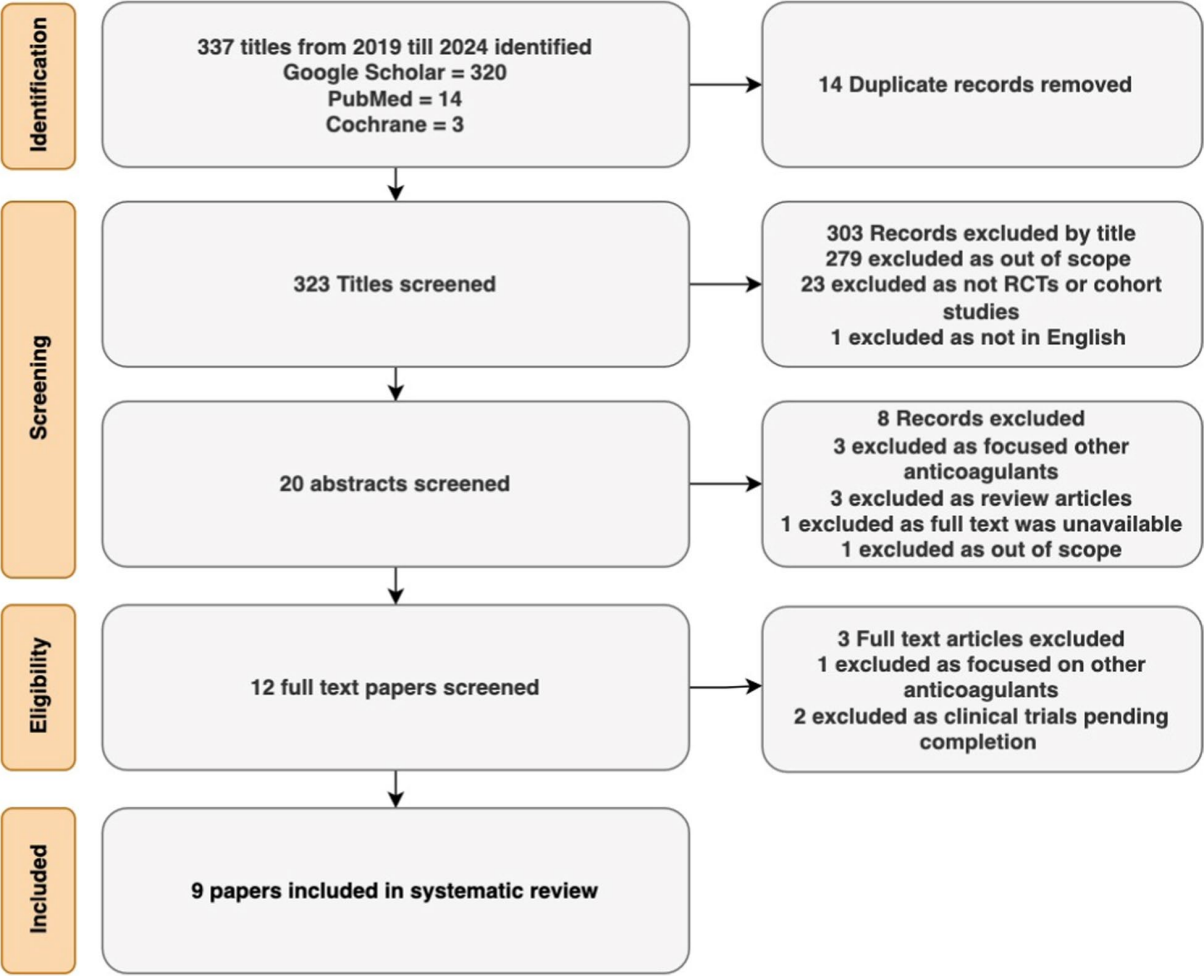
PRISMA flow diagram

The study selection process is illustrated in the PRISMA 2020 flow diagram (Fig. 1).

Nine studies were included in this systematic review and meta-analysis, three being prospective RCTs, three prospective cohort studies, two retrospective cohort studies and one being a retrospective cohort analysis of prospectively collected data. Each study had a similar intervention that being the administration of rivaroxaban, while comparisons were made with heparins, three studies did not have a control arm. A summary of the study characteristics can be found in Table 1.

**Table 1:** Study characteristics

First author, date, country	Title	Study design	Number of patients analysed	Type of surgery	Intervention	Comparison	Primary outcomes	Secondary outcomes
Kröll et al. [11], Switzerland	Efficacy and safety of rivaroxaban for postoperative thromboprophylaxis in patients after bariatric surgery: a randomized clinical trial	Prospective RCT	269	Roux-en-Y Gastric Bypass, Sleeve Gastrectomy, Revisional Surgery	Rivaroxaban	Not applicable	DVT, PE	Major or clinically relevant non-major bleeding and all-cause mortality
Eissa et al. [5], Egypt	Oral rivaroxaban for postoperative prophylaxis of venous thromboembolism after sleeve gastrectomy in patients with morbid obesity; is it safe: A prospective randomized clinical trial	Prospective RCT	500	Sleeve Gastrectomy	Rivaroxaban	Post-operative LMWH	VTE (DVT, PE, PMVT)	Bleeding
Farrag et al. [8], Egypt	Is rivaroxaban a safe and effective oral alternative to low-molecular-weight heparin in prophylaxis of portomesenteric and lower-limb deep-vein thrombosis after sleeve gastrectomy?	Prospective RCT	600	Sleeve Gastrectomy	Rivaroxaban	Post-operative LMWH	LL DVT and PMVT	Surgical complications, bleeding grade, recorded cases of Hb drop during follow-up CBC, abdominal bleeding or perigastric haematoma
Tyselskyi et al. [19], Ukraine	Prolonged thromboprophylaxis with rivaroxaban after bariatric interventions: A single-centre experience	Prospective cohort study	110	Sleeve Gastrectomy, Mini Gastric Bypass, Biliopancreatic Diversions	Rivaroxaban	Not applicable	Clinical or radiological VTE	Adverse events
Elemawy et al. [6], Egypt	Assessment of rivaroxaban as venous thromboembolism prophylaxis after gastric bypass surgery	Prospective cohort study	40	Antecolic Antegastric Roux-en-Y Gastric Bypass	Rivaroxaban	Not applicable	VTE	Intraluminal bleeding
Bayat et al. [4], Iran	The influence of enoxaparin or rivaroxaban for venous thromboembolism in morbidly obese patients undergoing bariatric surgery	Population-based, retrospective cohort study	1,000	Laparoscopic Sleeve Gastrectomy	Rivaroxaban	Post-operative LMWH	VTE	Not defined
Swartz et al. [18], USA	30-day post-discharge prophylaxis with rivaroxaban prevents porto-mesenteric venous thrombosis following laparoscopic sleeve gastrectomy	Retrospective cohort study	286	Laparoscopic or Robotic Sleeve Gastrectomy	Rivaroxaban	In-hospital heparins	PMVT	VTE and bleeding
Rodríguez et al. [16], Chile	Prophylaxis with rivaroxaban after laparoscopic sleeve gastrectomy could reduce the frequency of portomesenteric venous thrombosis	Retrospective cohort analysis of prospectively collected data	421	Laparoscopic Sleeve Gastrectomy	Rivaroxaban	In-hospital LMWH	PMVT or VTE	Bleeding
Ezz et al. [7], Egypt	A prospective study comparing the effect of rivaroxaban vs. enoxaparin given as a prophylaxis for deep venous thrombosis in patients undergoing bariatric surgery	Prospective study	40	Bariatric Surgery	Rivaroxaban	Post-operative LMWH	VTE (DVT)	Bleeding

**Fig. 2:**
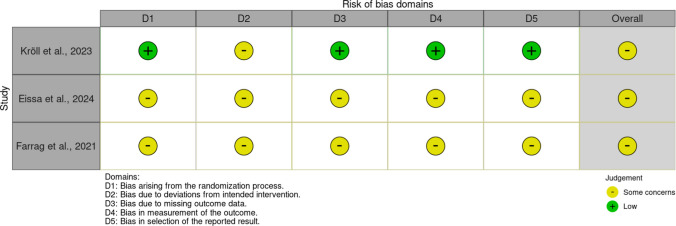
Visualisation of risk of bias results using Rob 2 for RCTs. For RoB 2, each domain was rated as "Low risk," "Some concerns," or "High risk" according to Cochrane guidance

**Fig. 3:**
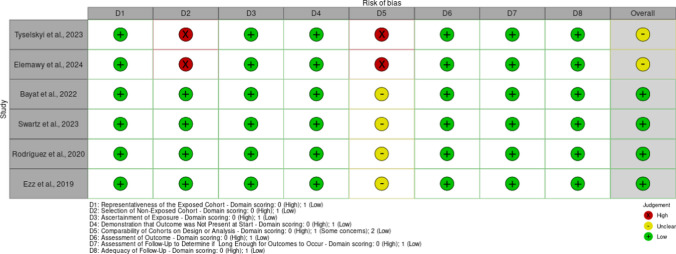
Visualisation of risk of bias results using NOS for cohort studies. Studies were classified as having a low risk of bias and high methodological quality if they scored 7 to 9 points on the NOS. A score of 5 to 6 points indicated moderate quality and was categorised as having some concerns regarding risk of bias. Studies scoring less than 5 points were considered to have a high risk of bias and low methodological quality

The risk of bias in the included studies was independently assessed by two reviewers using two validated tools: the Cochrane Risk of Bias 2 (RoB 2) tool for randomised controlled trials (RCTs), and the Newcastle–Ottawa Scale (NOS) for non-randomised studies. Visualisation of the risk-of-bias assessments was performed using the robvis tool as in Figs. 2 and 3 [14].

Statistical analysis for this review was conducted using RStudio, employing the metabin function from the meta package (version 8.0–2) to perform a meta-analysis of binary outcome data. A random-effects model was applied, risk difference was chosen as an effects measure, standard metrics were applied to assess for heterogeneity (I2, τ2, p-value). In the subgroup analysis, odds ratio (OR) was used.

## Results

From the total of 3,266 patients across the nine studies included, 1,856 patients were included in the rivaroxaban arm and 1,410 patients in the control arm. All patients in the rivaroxaban group received the 10 mg dose, except for a 100 patients in one study who received 2.5 mg twice daily instead, due to a lower risk of venous thromboembolism as assessed by the Caprini score [19]. Three studies did not include a control group hence the difference in numbers between the two arms [6], [11], [19]. The drug regimen used in the rivaroxaban and control arms are summarised in Tables 2 and 3. Studies were categorised according to treatment duration: short course (≤ 14 days) and extended course (> 14 days to ≤ 30 days) following initiation.

**Table 2:** Drug regimen used in rivaroxaban arm

	TOTAL (*N* =)	DOSE RECEIVED	STARTING POINT	DURATION (DAYS)
Kröll et al. [11]	269	10 mg daily	Day 1 post-op	7 (in 134 patients) 28 (in 135 patients)
Eissa et al. [5]	250	10 mg daily	Day 1 post-op	30
Farrag et al. [8]	300	10 mg daily	Day 1 post-op	14
Tyselskyi et al. [19]	110	2.5 mg BD (in 100 patients) 10 mg daily (in 10 patients)	Day 4 post-op	30
Elemawy et al. [6]	40	10 mg daily	24 h post-op	30
Bayat et al. [4]	500	10 mg daily	Day 1 of admission	Until hospital discharge
Swartz et al. [18]	144	10 mg daily	On discharge	30
Rodríguez et al. [16]	223	10 mg daily	On discharge	10
Ezz et al. [7]	200	10 mg daily	12 h post-op	14

**Table 3:** Drug regimen used in the control arm

	TOTAL (*N* =)	DOSE RECEIVED	STARTING POINT	DURATION (DAYS)
Kröll et al. [11]	0	-	-	-
Eissa et al. [5]	250	40 mg (Enoxaparin)	Day 1 post-op	30
Farrag et al. [8]	300	40 mg (BMI < 50 kg/m^2^), 60 mg (BMI ≥ 50 kg/m^2^) (Enoxaparin)	Day 1 post-op	14
Tyselskyi et al. [19]	0	-	-	-
Elemawy et al. [6]	0	-	-	-
Bayat et al. [4]	500	2.5 mg BD (Enoxaparin)	Day 1 of admission	Until hospital discharge
Swartz et al. [18]	142	5,000 Units (UFH)/ 40 mg (Enoxaparin)	6–12 h pre-op	In-hospital (3 post-op doses)
Rodríguez et al. [16]	198	40 mg (Enoxaparin)	Same day of operation	2 to 3
Ezz et al. [7]	20	1/2 mg/kg daily (Enoxaparin)	12 h post-op	14

Baseline patient characteristics show a female predominance in both groups. The mean age of patients in the rivaroxaban arm was 40.5 years while in the control arm was 39.7 years. The mean BMI was 46.3 kg/m^2^ in the rivaroxaban group and 45.3 kg/m^2^ in the control group. Of the total patients included across both arms, 3,005 underwent laparoscopic or robotic SG, 178 underwent a full gastric bypass, 23 had a mini-gastric bypass, 17 underwent revisional surgery, and 3 received a biliopancreatic diversion. In 40 patients, the type of bariatric surgery was not specified. These figures indicate that the studies predominantly represent patients undergoing SG. In four studies the recorded mean duration for bariatric surgeries was 85.5 min, while the mean for sleeve gastrectomies was 59.8 min. Documented duration of hospitalisation varied from one day up to three days post-operatively. Baseline patient characteristics and commonly reported comorbidities are summarised in Tables 4 and 5. A summary of the inclusion and exclusion criteria can be found in Table 6.

**Table 4:** Baseline patient characteristics in the rivaroxaban arm *In [8], the exact number of male and female participants was not specified. It was also the only study not to record the mean BMI as part of their patient characteristics

Baseline patient characteristics (Rivaroxaban arm)	Kröll et al. [11]	Eissa et al. [5]	Farrag et al. [8]	Tyselskyi et al. [19]	Elemawy et al. [6]	Bayat et al. [4]	Swartz et al. [18]	Rodríguez et al. [16]	Ezz et al. [7]
Male	53	125		29	13	185	28	82	9
Female	216	125		81	27	315	116	141	11
Age (years) (mean)	40	34.4 ± 6.41	35.01 ± 11.68	43.65	41.2 ± 9.86	45.2 ± 3.1	42.7	34.5	47.35 ± 13.92
Height, m (mean)	1.7				1.66 ± 0.08				
Weight, kg (mean)	117.8				131.3 ± 21.1		131.4		
BMI, kg/m2 (mean)	42.2	44.54 ± 3.37		55	47.81 ± 8.4	49.6 ± 5.6	48	35.7	47.82 ± 6.42
Comorbidities									
Heart disease	15								
Diabetes	33	40		29	27		28		
OSA	80						90		
HTN		10		19	21		74		
Asthma/COPD		20		7	3				
Chronic venous insufficiency of lower extremity				34					
Osteoarthritis					3				
Hyperlipidaemia					15		36		
GORD	79								
Smoker	79								
ASA score									
1	1								
2	68					500			
3	200

**Table 5:** Baseline patient characteristics in the control arm

Baseline patient characteristics (Control arm)	Eissa et al. [5]	Farrag et al. [8]	Bayat et al. [4]	Swartz et al. [18]	Rodríguez et al. [16]	Ezz et al. [7]
Male	85		205	32	63	10
Female	165		295	110	135	10
Age, years (mean)	35.7 ± 7.2	36.12 ± 12.34	43.8 ± 3.5	42.6	36.3	43.50 ± 11.34
Height, m (mean)						
Weight, kg (mean)						
BMI, kg/m2 (mean)	45.02 ± 3.43		47.2 ± 1.7	47.4	36.2	50.73 ± 14.81
Comorbidities						
Heart disease						
Diabetes	16			18.3		
OSA				52.8		
HTN	20			47.9		
Asthma/COPD	16					
Chronic venous insufficiency of lower limbs						
Osteoarthritis						
Hyperlipidaemia				16.9		
GORD						
Smoker						
ASA score						
1						
2			500			
3

**Table 6:** Summary of the inclusion and exclusion criteria of the included studies

	Kröll et al. [11]	Eissa et al. [5]	Farrag et al. [8]	Tyselskyi et al. [19]	Elemawy et al. [6]	Bayat et al. [4]	Swartz et al. [18]	Rodríguez et al. [16]	Ezz et al. [7]
Inclusion Criteria: Age	18 years or older	18–60 years	18 to 60 years	over 18 years	18–60 years	≥ 35 years	/	/	18 to 50 years
Inclusion Criteria: Sex	/	/	male or female	/	male and female	/	/	/	male and female
Inclusion Criteria: BMI	greater than 35 kg/m^2^	greater than or equal to 40 kg/m^2^ or more than 30 kg/m^2^ with comorbidities	more than 35 kg/m^2^ or more than 30 kg/m^2^ with comorbidities	above 35 kg/m^2^	greater than or equal to 40 kg/m^2^ or greater than 35 kg/m^2^ with obesity-related comorbidities	≥ 40 kg/m^2^	/	/	between 35 kg/m^2^ and 40 kg/m^2^ and other significant diseases that could be improved with weight loss
Inclusion Criteria: Other	failure of conservative treatment for 2 years	/	/	no evidence or past medical history of coagulopathy disorders	underwent gastric bypass surgery	/	meet standard criteria for bariatric surgery (as determined by the National Institutes of Health 1991 guidelines) and felt to be appropriate candidates for surgery	/	all appropriate non-surgical measures have been tried but the person has not achieved or maintained adequate, clinically benefical weight loss, the person commits to the need for long term follow up
Exclusion Criteria: Pregnant or breast feeding	pregnant or breastfeeding	pregnant or lactating	pregnant or lactating	pregnancy	/	/	/	/	/
Exclusion Criteria: Psychiatric disorder	/	psychiatric disorders	psychiatric disorders	mental impairment, non-compliance	major psychological disorders, drug and/or alcohol abuse patients	/	/	/	/
Exclusion Criteria: Drug allergy	/	known allergy to the studied drugs	known allergy to the studied drugs	/	/	/	/	/	/
Exclusion Criteria: Bleeding risk	had active bleeding or a high risk of bleeding	contraindication to anticoagulation, such as recent cerebral haemorrhage or coagulopathy	contraindication to anticoagulation, such as recent cerebral haemorrhage, active bleeding, coagulopathy, thrombocytosis	severe hyper- or hypocoagulation disorders	/	/	/	/	/
Exclusion Criteria: Thrombosis risk	history of VTE	/	known protein C, protein S, and antithrombin III deficiency	pre-existing VTE and anticoagulation for other reasons	/	/	on chronic anticoagulant medications	preoperative use of anticoagulation therapy	history of previous VTE
Exclusion Criteria: Peri-operative complications	/	patients converted to open technique due to intraoperative adverse events, major postoperative complications as reactionary bleeding or leakage	laparoscopic sleeve converted to open, developed surgical complications, such as bleeding in the first 24 h after surgery, which required surgical interventions	/	/	/	/	/	/
Exclusion Criteria: Other surgeries	/	history of previous bariatric surgeries	previous bariatric surgeries, patients who underwent one anastomosis gastric bypass	/	previous bariatric surgery	/	/	/	/
Exclusion Criteria: Other comorbidities	/	/	/	age under 18 years, poorly controlled diabetes (> 9 mmol/l)	surgically unfit patients with compromised cardiopulmonary function, chronic decompensated diseases (renal or hepatic), incurable cancer, secondary obesity (hormonal disturbances)	/	/	impaired renal function clearance (creatinine clearance < 50 ml/minute)	history of cardiac or chest disease, patient on hormone replacement therapy or oral contraceptive pills

This review also evaluated the duration and adequacy of postoperative follow-up (Table 7), including the post-operative investigations performed and the diagnostic methods used to identify both primary and secondary outcomes (Table 8), in order to assess the appropriateness of follow-up protocols.

**Table 7:** Planned duration of patient follow-up in the included studies

	Kröll et al. [11]	Eissa et al. [5]	Farrag et al. [8]	Tyselskyi et al. [19]	Elemawy et al. [6]	Bayat et al. [4]	Swartz et al. [18]	Rodríguez et al. [16]	Ezz et al. [7]
Length of follow-up	28 ± 2 days	30 days	1 month	6 months	1 month	3 months	30 and 90 days	24 months	3 months

**Table 8:** Follow-up methods described in included studies

	Pre-operative assessment	Blood test follow-up	Ultrasound follow-up for DVT	Timing of ultrasound follow-up	Clinical-follow up	Timing of clinical follow-up	PE diagnostics	PMVT diagnostics	Bleeding diagnostics
Kröll et al. [11]	/	/	bilateral compression U/S screening of both legs	after 28 days (with a window of ± 2 days)	safety follow-up visit was performed by telephone	at day 35 (1 week after the U/S assessment)	based on clinical signs and symptoms during hospitalisation and follow- up; if suspected, confirmed by contrast-enhanced spiral CT or ventilation/perfusion scintigraphy	/	/
Eissa et al. [5]	/	CBC	suspected cases of LL DVT assessed with duplex U/S	/	/	/	/	suspected portal vein thrombosis were evalutated using CT abdomen with IV contrast	CT abdomen + IV contrast in clinically suspected cases where U/S was inconclusive or showed suspected intraabdominal collection
Farrag et al. [8]	/	CBC	no need for LL venous duplex as none were clinically detected	/	/	/	/	routine U/S was done 3 days post sleeve gastrectomy even if the patient had no complaints	follow-up U/S
Tyselskyi et al. [19]	pre-operative U/S of the veins of the lower extremities, portal vein and great vessels	/	U/S examination of the portal vein and the veins of the lower extremities	3rd, 30th and 60th post-operative days	telephone interviews to evaluate the presence of complaints which may indicate VTE and to assess the patients’ compliance with the regimen and satisfaction	30 and 60 days after the surgery	16 CTPA scans performed based on respiratory complaints	the patients underwent U/S of the portal vein and the veins of the lower extremities on the 3rd, 30th and 60th day after the operation	/
Elemawy et al. [6]	full history, clinical examination, anthropometric measurements, lab investigations, imaging (pelvic-abdominal U/S), chest X-ray, respiratory function tests, echo, ECG, and pulmonary function tests, and OGD when indicated	/	/	/	/	/	/	/	clinical presentation with melena, tachycardia, and hypotension
Bayat et al. [4]	/	/	/	/	/	/	/	/	/
Swartz et al. [18]	/	/	/	/	clinical follow-up	one week post-op in the non rivaroxaban group, follow-up data at 30 and 90 days; if no follow-up records, patients were contacted by telephone to complete the data questionnaire	/	CT scan when symptomatic —performed in 33 patients in the rivaroxaban group	/
Rodríguez et al. [16]	/	/	/	/	clinical follow-up	10 days after the surgery, at the first month after surgery, then every 3 months during the first year and every 4 months during the second year	/	contrast-enhanced CT for every patient who presented to ED with abdominal pain in the post-operative period	/
Ezz et al. [7]	history, general and local examination, blood investigations	/	venous duplex on the lower limbs regularly as screening for clinical or subclinical DVT	1 and 3 months after surgical intervention	followed up clinically as screening for clinical and subclinical DVT	1 and 3 months after surgical intervention	/	/	/

Out of a total of 1,856 patients in the rivaroxaban arm, symptomatic or asymptomatic DVT was reported in four studies, with [7] noting the highest number (n = 2), while [4] and [11] each reported a single case. PE was documented only in one study [18], and PMVT was not observed in any of the studies (Table 9).

**Table 9:** Summary of the results of the primary and secondary outcomes in the rivaroxaban arm

	SYMPTOMATIC/ASYMPTOMATIC DVT	PE	PMVT	MINOR BLEEDING/GRADE 1	MODERATE-TO-MAJOR/GRADE 2	MAJOR REQUIRING/GRADE 3	MAJOR REQUIRING TRANSFUSION/GRADE 3
Kröll et al. [11]	1	0	-	10	3	0	2
Eissa et al. [5]	0	0	0	41	1	0	0
Farrag et al. [8]	0	-	0	40	1	0	1
Tyselskyi et al. [19]	0	0	0	1	0	0	0
Elemawy et al. [6]	0	0	-	0	0	0	3
Bayat et al. [4]	1	-	-	-	-	-	-
Swartz et al. [18]	0	1	0	2	0	2	0
Rodríguez et al. [16]	0	0	0	0	0	0	0
Ezz et al. [7]	2	-	-	0	0	0	0

Among the thromboembolic events reported in the rivaroxaban arm, one patient in the study by [11] was diagnosed with asymptomatic DVT following a SG while on an extended course of thromboprophylaxis. In contrast, three patients in the studies by [4] and [7] developed DVT while receiving short-course anticoagulation. The patient in [4], a male with a history of pulmonary thromboendarterectomy, underwent SG and received thromboprophylaxis from admission until discharge. He developed DVT 23 days following discharge. In [7] the two patients who underwent bariatric surgery receiving rivaroxaban for 14 days, starting 12 h postoperatively, one developed DVT at one month and the other at three months postoperatively. The only PE reported in the rivaroxaban group occurred in [18], where the patient underwent laparoscopic SG and received 30 days of rivaroxaban. He developed this on postoperative day 45, fifteen days after the completion of thromboprophylaxis.

Minor bleeding (Grade 1) was the most frequently observed adverse event. [5] reported 41 cases, including 37 port-site haematomas and four instances of menorrhagia. [8] documented 40 cases, all of which involved petechial bleeding. [11] noted 10 cases, and [18] reported two cases of small haematomas. Additionally, [19] reported one case of a mild subcutaneous haematoma of the anterior abdominal wall, however, it was not specified whether the patient received the 10 mg once-daily or the 2.5 mg twice-daily dose of rivaroxaban. No such events were reported in the remaining studies. Moderate-to-major bleeding (Grade 2) occurred in three patients in [11], and this was categorised as clinically relevant non-major bleeding. Another case of perigastric haematoma occured in each study by [5] and [8]. Major bleeding requiring surgical intervention (Grade 3) occurred only in one study by [18] which reported two cases namely a staple line bleed and a rectus sheath hematoma. Major bleeding requiring transfusion was reported in [11] with two cases of major bleeding events, [8] reported one case of haematemesis managed with blood transfusion and oesophagogastroduodenoscopy (OGD) and [6] documented three cases of intraluminal bleeding. Overall, thromboembolic events were infrequent, while minor bleeding complications were more commonly encountered across the included studies (Table 9).

Among the 1,410 patients in the control group, DVT was reported in three patients in the study by [7]. PE was reported in a single patient in [18]. PMVT was observed across three studies: one case in [5], and four cases each in [16] and [18] (Table 10).

**Table 10:** Summary of the results of the primary and secondary outcomes in the control arm

	SYMPTOMATIC/ASYMPTOMATIC DVT	PE	PMVT	MINOR BLEEDING/GRADE 1	MODERATE-TO-MAJOR/GRADE 2	MAJOR REQUIRING SURGERY/GRADE 3	MAJOR REQUIRING TRANSFUSION/GRADE 3
Kröll et al. [11]	-	-	-	-	-	-	-
Eissa et al. [5]	0	0	1	29	2	0	1
Farrag et al. [8]	0	-	0	1	12	0	0
Tyselskyi et al. [19]	-	-	-	-	-	-	-
Elemawy et al. [6]	-	-	-	-	-	-	-
Bayat et al. [4]	0	-	-	-	-	-	-
Swartz et al. [18]	0	1	4	2	0	5	0
Rodríguez et al. [16]	0	0	4	0	0	0	0
Ezz et al. [7]	3	-	-	0	0	0	0

Within the control group, one study reported three cases of DVT in patients who had undergone bariatric surgery while receiving a 14 day course of enoxaparin [7]. DVT was diagnosed in one patient at one month postoperatively and in two others at three months postoperatively. A single case of PE was reported in [18], however, the specific type of thromboprophylaxis administered to this patient was not documented. In this study, patients received either UFH, LMWH, or in a few cases, no anticoagulation.

PMVT represented the most frequently reported thromboembolic complication in the control group, with a total of nine cases across studies. One patient, who was receiving an extended course of LMWH, presented with PMVT 20 days postoperatively [5]. The remaining eight cases, four in [18] and four in [16], occurred in patients who had only received in-hospital thromboprophylaxis (short-course regimen). Among the latter cohort, patients were aged 44, 35, 19, and 28 years, three of whom were female, with a mean BMI of 35.2 kg/m2. Common comorbidities included insulin resistance, diabetes mellitus and dyslipidaemia, and with documented habits of tobacco and/or alcohol use. The average operative time was 66 min, and hospital stays ranged from two to three days. Notably, the youngest patient, a female aged 19, was using the oral contraceptive pill and was later found to have protein S deficiency upon thrombophilia testing. The latter, along with one other patient, exhibited the most extensive thrombotic involvement, with propagation to the main portal vein, right and left portal veins, splenic vein, and superior mesenteric vein.

Minor bleeding events (Grade 1) were most commonly reported in [7], affecting 29 patients, 26 of whom experienced port-site haematomas and three who reported menorrhagia. [8] documented one case of petechial bleeding in a patient receiving LMWH, although the specific dose was not provided, as dosing in this study was based on BMI. [18] reported two additional cases of minor bleeding. Moderate-to-major bleeding events (Grade 2) were most frequently observed in [8], where 12 patients developed perigastric haematomas and were classified within this subcategory. Of these, 10 were managed conservatively, while the remaining two required laparoscopic evacuation due to their size, thus overlapping with the category of major bleeding requiring surgical intervention (Grade 3). Notably, one of these cases was complicated by haematoma infection and subsequent leakage, necessitating the placement of a mega-stent. [5] reported two additional cases of perigastric haematomas. Major bleeding requiring surgery (Grade 3) was reported solely by [18]. In this study, five patients underwent surgical intervention: four returned to theatre within four days following SG, two for staple-line bleeding and two for haematoma evacuation with no identifiable bleeding source. The fifth patient developed a massive PMVT and splenic vein thrombosis complicated by splenic rupture and acute haemorrhage on postoperative day fifteen. Major bleeding events requiring transfusion were rare, with only one instance reported in [5], involving a patient with haematemesis and melena necessitating blood transfusion (Table 10).

**Fig. 4:**
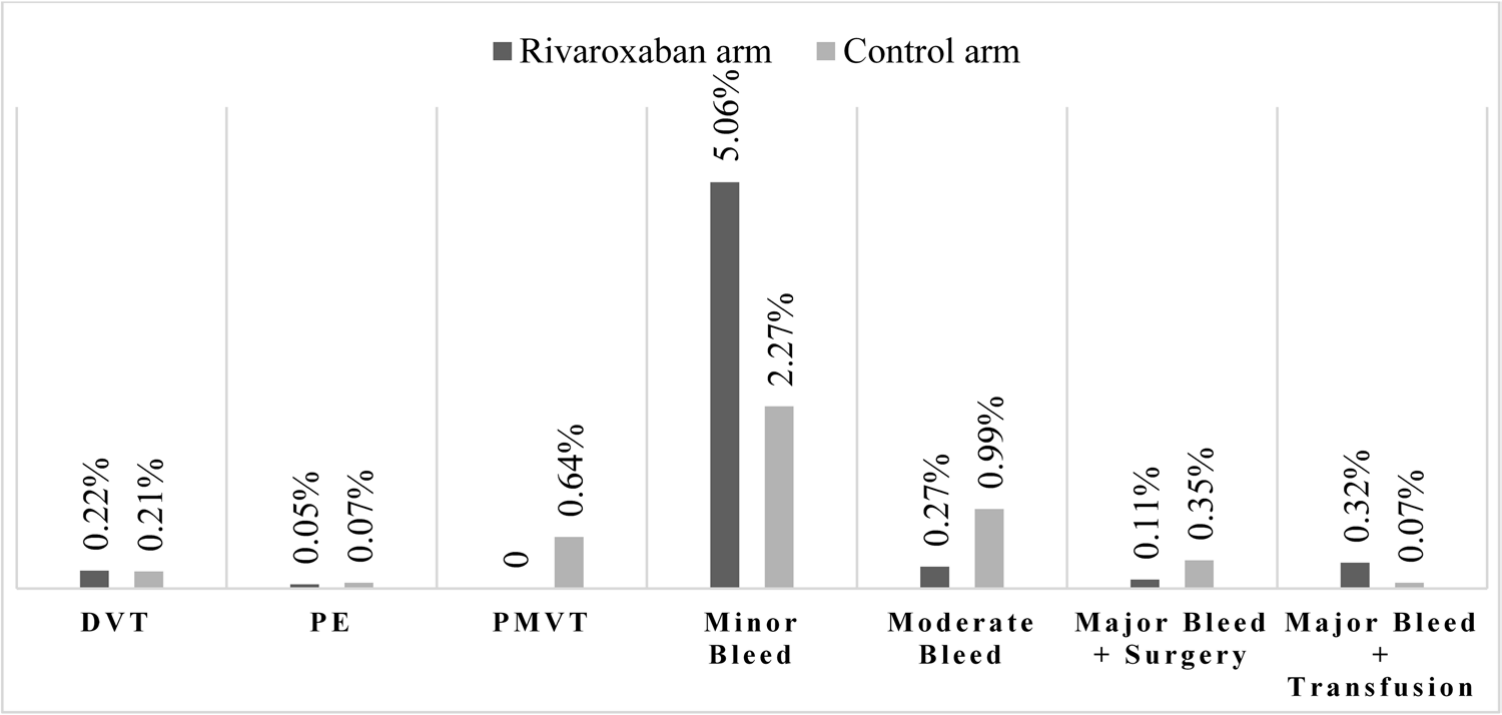
A comparative descriptive analysis of adverse events between the rivaroxaban and control arms across several outcome categories, expressed as percentages of the total number patients in each group (n = 1859 in rivaroxaban arm and n = 1410 in control arm)

The comparative descriptive analysis between the rivaroxaban and control arms (Table 11, Fig. 4) revealed similar rates of DVT occurring in 0.22% of patients in the rivaroxaban group (n = 4) and 0.21% in the control group (n = 3). PE was slightly less frequent in the rivaroxaban group (0.05%, n = 1) compared to the control group (0.07%, n = 1). Notably, PMVT was not observed in any patients receiving rivaroxaban, whereas it was reported in 0.64% of patients in the control arm (n = 9), suggesting a potential advantage of rivaroxaban in preventing this complication.

**Table 11:** A comparative descriptive analysis of adverse events between the rivaroxaban and control arms across several outcome categories, expressed as number of patients

	Symptomatic/Asymptomatic DVT	PE	PMVT	Minor bleeding/Grade 1	Moderate-major/Grade 2	Major bleeding requiring surgery/Grade 3	Major requiring transfusion/Grade 3
Rivaroxabanarm	4	1	0	94	5	2	6
Control arm	3	1	9	32	14	5	1

In terms of bleeding events, minor bleeding (Grade 1) was more common in the rivaroxaban group, affecting 5.06% of patients (n = 94), compared to 2.27% in the control group (n = 32). Conversely, moderate-to-major bleeding (Grade 2) occurred more frequently in the control arm (0.99%, n = 14) than in the rivaroxaban arm (0.27%, n = 5). Major bleeding requiring surgical intervention (Grade 3) was slightly more frequent in the control group (0.35%, n = 5) than in the rivaroxaban group (0.11%, n = 2). Major bleeding events requiring blood transfusion were more frequent in the rivaroxaban arm (0.32%, n = 6) compared to the control arm (0.07%, n = 1). Overall, rivaroxaban demonstrated comparable efficacy in preventing DVT and PE and was associated with a complete absence of PMVT in the included population. While the rates of minor bleeding and major bleeding requiring transfusion were more common with rivaroxaban, the incidence of moderate bleeding and major bleeding requiring surgery were lower.

## Meta-analysis of Risk Differences

**Fig. 5:**
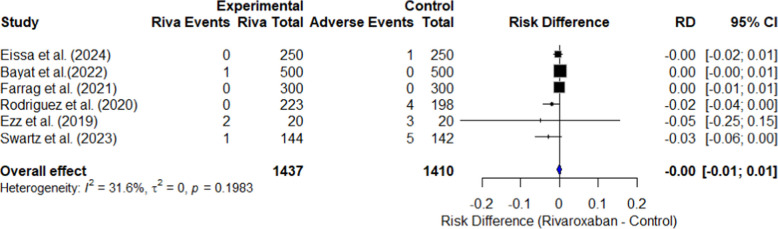
Forest plot comparing rivaroxaban and control groups for thrombotic adverse events as primary outcomes in thromboprophylaxis following bariatric surgery

The primary outcome combined forest plot (Fig. 5), which includes the studies with a control arm, shows that the use of rivaroxaban for thromboprophylaxis following bariatric surgery has no difference in the risk of adverse events compared to control interventions. The pooled RD was 0.00 (95% CI: −0.01 to 0.01), indicating clinical equivalence between the two groups. Furthermore, heterogeneity among the included studies was low to moderate (I2 = 31.6%) and not statistically significant (p = 0.1983), supporting the robustness of the overall findings.

A notable observation in the study by [7], is the relatively high number of reported adverse events in both the rivaroxaban and control groups, despite the small overall sample size [7]. Larger sample sizes are generally preferred in such studies, as the limited number of participants here makes it difficult to determine whether rivaroxaban or the control treatment has a definitive impact on DVT as an adverse outcome.

**Fig. 6:**
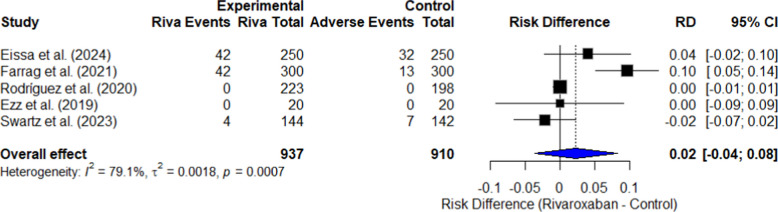
Forest plot comparing rivaroxaban and control groups for bleeding adverse events as secondary outcomes in thromboprophylaxis following bariatric surgery

The forest plot in Fig. 6 illustrates a meta-analysis comparing the incidence of bleeding events between patients receiving rivaroxaban and those in the control group as thromboprophylaxis following bariatric surgery. The pooled analysis includes data from five studies, comprising 937 patients in the rivaroxaban group and 910 in the control group. The overall RD was 0.02 with a 95% confidence interval (CI) of −0.04 to 0.08. This indicates that whilst the studies seem to suggest slightly higher bleeding events in the rivaroxaban group and a better positive outcome with the control group, this cannot be considered significant evidence against its use. Substantial heterogeneity among the studies (I2 = 79.1%, p = 0.0007), indicates considerable variability in effect sizes across the included trials further limiting the strength of this outcome as an argument against rivaroxaban use. Despite what appears to be an increase in bleeding risk with rivaroxaban, this meta-analysis could only serve as a limited argument against its use. The increased bleeding rates observed in the rivaroxaban group are likely influenced by random variation or other uncontrolled factors rather than a definitive drug-related effect. Therefore, rivaroxaban should not be considered significantly worse than alternative medications based on the current evidence, when considering bleeding alone.

## Subgroup Analysis

**Fig. 7:**
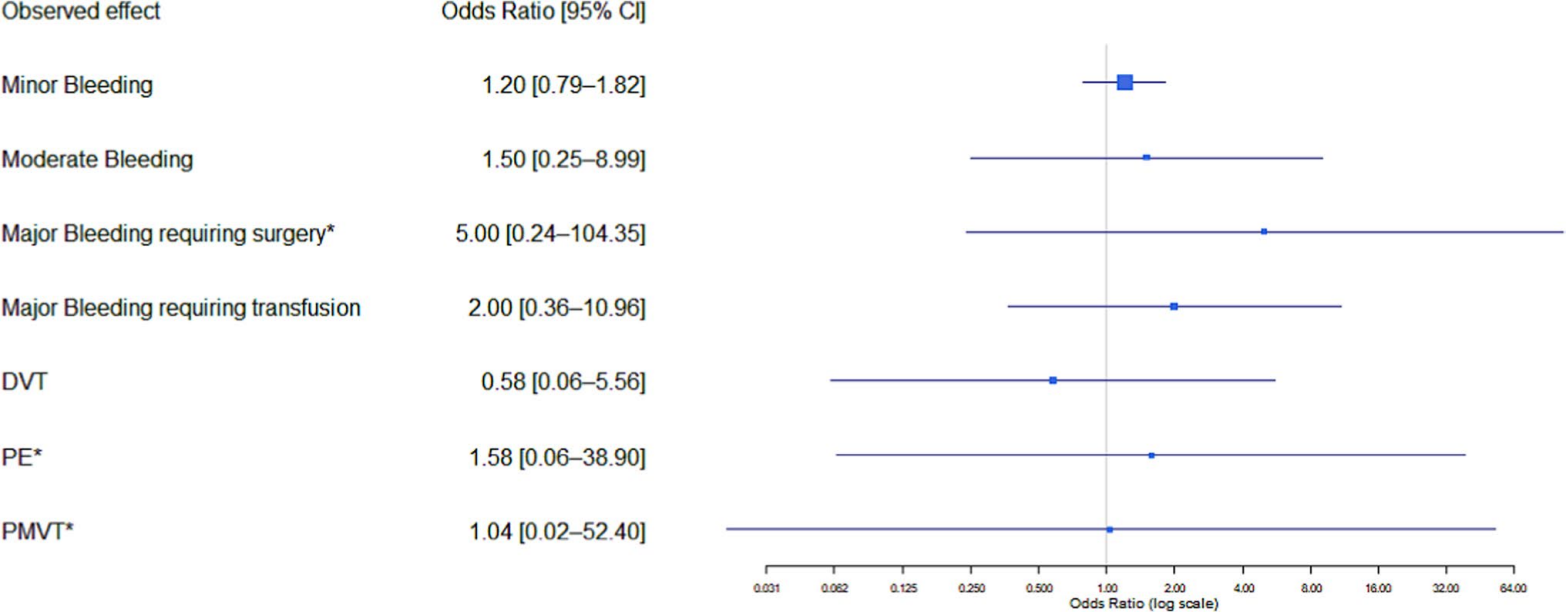
Forest plot comparing long-course to short-course duration of rivaroxaban for thromboprophylaxis after bariatric surgery showing ORs with 95% CIs for various adverse outcomes. An OR > 1 suggests the event is more likely with long-course prophylaxis; OR < 1 suggests the event is more likely in the short-course group. (* Modified Haldane-Anscombe (mHA) corrections were made only to the crosstabulations that had 0 in one of their cells and are marked with an asterisk [20].)

A subgroup analysis was conducted to compare thromboembolic outcomes between long-duration and short-duration rivaroxaban prophylaxis regimens (Fig. 7).

A comparison of adverse events between long and short courses of rivaroxaban revealed no statistically significant differences across all outcomes. For DVT, the OR was 0.58 (95% CI: 0.06–38.90), with a p-value of 1.00, suggesting a lower, and possible protective effect with long-course prophylaxis however results are inconclusive due to a wide CI. For PE, the OR was 1.58 (95% CI: 0.06–38.90), with a p-value of 0.78 using the mHA correction, with one event observed only in the long-course group. No PMVT events occurred in either group (OR of 1.04 using the mHA correction (95% CI: 0.02–52.40), with a p-value of 0.99), therefore there were no differences between the two groups. These findings indicate that extending the duration of rivaroxaban therapy did not significantly alter the risk of thromboembolic complications in this population. One may also conclude that a prolonged duration of rivaroxaban may not substantially reduce thromboembolic risk compared to a shorter course, indicating that shorter prophylaxis regimens may be adequate for most patients following bariatric surgery. However, the low event rates limit the statistical power and generalisability of these results, and further studies with larger sample sizes are warranted.

A subgroup analysis was performed to evaluate bleeding outcomes in relation to the duration of rivaroxaban prophylaxis. It is important to note that the study by [4] did not report on bleeding events, consequently, the 500 patients from that study were excluded from the total sample size used in analyses of bleeding events. The ORs for various bleeding complications were calculated to assess the comparative safety of long versus short courses of rivaroxaban following bariatric surgery. Minor bleeding was approximately 20% more likely in patients receiving a long course of rivaroxaban compared to those on a short course (OR of 1.20 (95% CI: 0.79–1.82), with a p-value of 0.45), although this difference was not statistically significant suggesting the result may be due to chance. Moderate bleeding shows a trend toward more bleeding with longer duration of prophylaxis (OR of 1.50 (95% CI: 0.25–8.99), with a p-value of 1.00). For major bleeding requiring surgical intervention, the OR was 5.00 using the mHA correction (95% CI: 0.24–104.35), with a p-value of 0.30, indicating that the risk appears substantially higher in the long-course group, but because of the small number of events, the result did not reach statistical significance. Similarly, major bleeding requiring transfusion was less common in the short-course group, with twice the odds in the long-course group (OR of 2.00 (95% CI: 0.36–10.96), with a p-value of 0.69), though the result also did not support a significant effect. Overall, while there appears to be a trend toward fewer severe bleeding events with shorter courses of rivaroxaban, none of the observed differences can be considered as significant evidence. These findings highlight the need for larger, adequately powered studies to clarify the optimal duration of thromboprophylaxis in this patient population.

These results may show that extended prophylaxis with rivaroxaban may be associated with a higher incidence of clinically significant bleeding events, including those requiring surgical intervention or blood transfusion. Although these findings did not reach statistical significance, the observed trend indicates that shorter courses of rivaroxaban may offer a more favorable bleeding profile, particularly in clinical scenarios where prolonged anticoagulation is not clearly indicated.

## Discussion

To our knowledge this is the first systematic review and meta-analysis of RCTs and cohort studies that focuses on rivaroxaban use as thromboprophylaxis following bariatric surgery. Given that the oral route is the preferred anticoagulation method, analysing data to assess the efficacy and safety of rivaroxaban as the main thromboprophylaxis treatment following bariatric surgery is of clinical relevance [19]. This systematic review addresses a significant gap in research, providing a deeper understanding of the optimal thromboprophylaxis strategy while balancing the risks of thromboembolism and bleeding which are the key challenges in post-bariatric care.

The overall incidence of primary endpoint events was low across the included studies, consistent with literature reporting VTE rates of 0.2%–5% and PMVT rates of 0.3%–1.8%, with PMVT being more prevalent following SG than other bariatric procedures [18], [19].

The meta-analysis findings suggest that rivaroxaban is not associated with any difference in thromboembolic risk relative to standard thromboprophylaxis regimens, thereby supporting its non-inferiority in the context of short-term prophylaxis. Notably, the absence of PMVT cases in the rivaroxaban arm may suggest a potential protective benefit that warrants further exploration. The incidence of PMVT was particularly elevated in studies where patients in the control arm received only in-hospital heparin [16, 18]. Several factors may account for this finding. Smaller sample sizes can inflate the apparent incidence of rare events, while the type of bariatric procedure, particularly the higher risk associated with laparoscopic sleeve gastrectomy may also contribute to increased rates. Additionally, surgeon experience has also been suggested as a potential risk factor. Interestingly, portal venous system thrombosis (PVST) is often asymptomatic or transient and is frequently identified incidentally through imaging. As a result, the true incidence of PVST in the postoperative setting may be underestimated in the certain studies [12].

While the analysis seems to suggest a slightly higher bleeding rate in the rivaroxaban group (RD 0.02 95% CI: –0.04 to 0.08), this cannot be considered significant evidence against its use. This finding must be interpreted with caution due to substantial heterogeneity across studies. Differences in definitions of bleeding severity, patient characteristics, and perioperative protocols contribute to variability in outcomes. The variability observed across studies may be attributed to several factors, including differences in study design, such as the choice of control groups, duration of follow-up, and surgical procedures employed. Additionally, concurrent use of other medications or advancements in surgical techniques could diminish the observable effect of rivaroxaban or the control intervention, although assuming this impact is equal across both arms may not hold true in all cases. Variations in patient populations and sample sizes, rivaroxaban dosing protocols, and the definitions or thresholds used to classify bleeding events further contribute to the heterogeneity of outcomes, complicating direct comparisons and pooled analyses.

Two studies reported a notably higher incidence of minor bleeding compared to the rest, raising the possibility that such events may have been underreported in other studies, or that specific factors unique to these two studies may have contributed to an increased risk of minor bleeding [5], [8]. Moreover, the absolute increase in bleeding risk remains modest. In clinical practice, the decision to use rivaroxaban should therefore be individualised, particularly in patients with elevated bleeding risk, with careful consideration of the overall risk–benefit profile.

The bleeding risk identified in this meta-analysis is consistent with previously published data, including pooled analyses from the RECORD trials. These trials, which evaluated rivaroxaban versus enoxaparin in patients undergoing total hip and knee replacement, demonstrated that rivaroxaban significantly reduced the incidence of symptomatic VTE and all-cause mortality during the study period. However, the rate of major bleeding was marginally higher in the rivaroxaban arm (0.39%) compared to the enoxaparin arm (0.21%), although this difference was not statistically significant (P = 0.076). Notably, the combined rate of major and clinically relevant non-major bleeding was significantly elevated with rivaroxaban [17].

Subgroup analysis comparing long-course versus short-course rivaroxaban regimens revealed no significant difference in thromboembolic outcomes between the two strategies. These results suggest that shorter courses of prophylaxis with rivaroxaban may be sufficient for most patients undergoing bariatric surgery. However, despite the lack of statistical significance, there was a trend toward increased rates of bleeding in the extended therapy group. This finding lends additional support to the use of shorter rivaroxaban regimens, particularly in the absence of specific indications for prolonged prophylaxis. These findings align with those of a recent clinical trial, which concluded that a 7-day course of prophylaxis following bariatric surgery may be adequate to prevent thromboembolic events in the majority of patients at moderate risk of VTE [11].

## Limitations

The current body of evidence supporting the use of rivaroxaban for thromboprophylaxis following bariatric surgery remains limited by the scarcity of high-quality RCTs and robust observational studies. This paucity raises concerns regarding potential publication bias and the underreporting of neutral or adverse outcomes. RCTs evaluating postoperative anticoagulation are inherently complex due to ethical considerations, and blinding can be particularly challenging when the intervention and control differ in route of administration, potentially introducing performance and detection bias.

Furthermore, the overall low incidence of thromboembolic and bleeding events across studies significantly reduces statistical power, limiting the ability to detect meaningful differences between treatment regimens and increases the chances of type two errors. Substantial heterogeneity across studies further complicates interpretation and synthesis. Heterogeneity in patient demographics (including: BMI, ASA classification, and comorbid conditions, baseline VTE and bleeding risk), surgical practice (including: surgical technique, surgeon experience and institutional perioperative protocols), operative duration, length of hospital stay, and inclusion/ exclusion criteria in the studies introduces significant confounding variability. Inconsistencies in follow-up intervals, diagnostic strategies, as well as in the timing of rivaroxaban initiation and its duration, limit the comparability of outcomes and preclude robust subgroup or stratified analyses. Discrepancies in diagnostic accuracy especially given the technical limitations of ultrasonography in patients with obesity and the short duration of follow-up in some studies may result in underreporting of delayed VTE or bleeding events.

Additional challenges arise from inconsistent outcome reporting, particularly regarding thromboembolic and bleeding events. These factors collectively hinder the feasibility of conducting a robust meta-analysis and highlight the need for standardised reporting protocols in future clinical trials.

Some studies employed bridging strategies using LMWH or UFH before transitioning to rivaroxaban, while others initiated rivaroxaban post-discharge. Bridging with a short-acting anticoagulant such as LMWH may be a more cautious strategy in the immediate postoperative period, followed by delayed initiation of rivaroxaban once the risk of early surgical complications has diminished in high risk patients. This approach aims to mitigate the heightened bleeding risk associated with early administration of rivaroxaban while still providing effective thromboprophylaxis during the vulnerable postoperative phase. One of the key concerns with initiating rivaroxaban immediately after surgery is the potential need for early re-intervention. This risk is especially pertinent during the first few postoperative days, a period during which complications such as anastomotic leaks, intra-abdominal haemorrhage, or wound dehiscence are most likely to arise and may require prompt surgical attention. Although rivaroxaban reaches peak plasma levels within 2 to 4 h, steady-state plasma concentrations, representing the full clinical anticoagulant effect, are consistently established by the fourth day of once-daily dosing [10]. As such, early postoperative use of rivaroxaban may result in maximal anticoagulant effect coinciding with the period when surgical complications typically manifest, thereby increasing the risk of bleeding if re-intervention becomes necessary. The management of such scenarios is further complicated by the absence of routine monitoring parameters for rivaroxaban and the limited availability of rapid reversal agents. This variation warrants further investigation to evaluate the comparative safety and efficacy of bridging versus direct initiation strategies.

Finally, ethical and safety considerations, particularly concerning bleeding risk with rivaroxaban, may limit the generalisability of findings to broader patient populations. The lack of long-term follow-up data further obscures the sustained efficacy and safety profile of rivaroxaban in this context. In view of the noted limitations and the uncertainty surrounding the available evidence, establishing the most effective and safest thromboprophylaxis regimen following bariatric surgery remains challenging. Findings in this study call for further high-quality, adequately powered RCTs to guide future clinical guidelines and define the optimal dosing strategy and duration that maximizes efficacy while minimising harm. Future research should also look further into the different bariatric procedures beyond SG, as surgical variations may affect bleeding and thrombotic risks, drug absorption, and the effectiveness of thromboprophylaxis.

In conclusion, rivaroxaban can be considered a safe and effective option for thromboprophylaxis following bariatric surgery, with short-course regimens being generally sufficient for low-to-moderate risk patients. Prolonged use should be reserved for selected high-risk patients, after a careful evaluation of the individual risk-benefit profile. Given the current limitations and uncertainty in the available evidence, determining the optimal thromboprophylaxis regimen for all bariatric patients continues to pose a significant clinical challenge.

## Data Availability

We have used research data that is publicly available on open-access websites.
